# Predicting Protein–Protein Interactions by Convolutional Neural Network Model

**DOI:** 10.3390/biotech15010020

**Published:** 2026-02-16

**Authors:** Shuaibo Shi, Ting Xiong, Dong Wang, Lingling Wei, Lin Li, Zhixin Li, Yanfen Lyu

**Affiliations:** 1 School of Mathematics and Physics, Hebei University of Engineering, Handan 056038, China; shi258329@163.com (S.S.);; 2 Zhaoqing Branch of Guangdong Laboratory of Lingnan Modern Agricultural Science and Technology, Zhaoqing 526238, China; bearvet@163.com; 3 School of Mechanical and Equipment Engineering, Hebei University of Engineering, Handan 056038, China; 4 College of Management Engineering and Business, Hebei University of Engineering, Handan 056038, China; 5 College of Chemical Engineering and Biotechnology, Xingtai University, Xingtai 054001, China

**Keywords:** protein–protein interactions, sample feature grayscale map, CNN

## Abstract

The study of protein–protein interactions (PPIs) is of significant importance for elucidating biological processes, clarifying pathological mechanisms, and promoting drug development. In this study, we proposed a method to predict PPIs based on protein sequence and gene sequence information, combined with convolutional neural networks (CNNs). First, we extracted three types of features from protein sequence: global physicochemical properties features of the protein sequence, local same type of amino acid position variation features, and protein evolutionary conservation features; simultaneously, we extracted single nucleotide frequency and positional features, dinucleotide frequency features, and trinucleotide frequency features from the corresponding gene sequence. During the feature extraction process, we employed the amphiphilic pseudo amino acid composition (APAAC) method to extract the global hydrophobicity and hydrophilicity features of the protein sequence; we defined a new mathematical descriptor—θ interval deviation product factor—to extract protein evolutionary conservation features from Position Specific Scoring Matrix (PSSM); we also defined a mapping function to map all nucleotides in the gene sequence onto a unit circle, and then extracted nucleotide positional features from the mapped points. Second, based on extracted features, we constructed a 36 × 32 sample feature grayscale map to represent a protein pair sample. Finally, we developed a CNN model to predict PPIs. Our method achieved superior results on four species test sets: an accuracy of 99.28% on the *Saccharomyces cerevisiae* dataset, 98.15% on the *Drosophila melanogaster* dataset, 98.62% on the *Homo sapiens* dataset, and 96.84% on the *Mus musculus* dataset, outperforming existing computational methods. Furthermore, we extended the application of this method to the prediction of protein–protein interaction networks and non-interaction networks, and also achieved promising results.

## 1. Introduction

Cells are the basic structural and functional units of living organisms, and proteins are important molecules that carry out various biological processes in life systems [1,2]. Although proteins are involved in regulating almost all cellular activities, they usually do not function in isolation. Most proteins form complex networks by interacting with other molecules (such as other proteins, DNA, RNA, metal ions, etc.) to coordinate life processes, including signal transduction, DNA replication, cell cycle progression, and enzyme-catalyzed reactions [3]. Studies have shown that abnormal PPIs are closely associated with some human diseases, including cancer [4], neurological disorders [5], immune dysfunction [6], bipolar disorder [7], and cardiovascular diseases [8]. Therefore, the study of PPIs not only helps to elucidate the mechanisms underlying these interactions and the pathogenesis of diseases but also provides a theoretical basis for the early diagnosis and treatment strategies of diseases.

Experimental methods for identifying PPIs include Co-Immunoprecipitation (Co-IP) [9], Yeast Two-Hybrid (Y2H) system [10], Protein Microarray technology [11], Protein Cross-Linking technology [12], and so on. Each of these methods has distinct characteristics: the Co-IP method is suitable for detecting PPIs with strong binding forces but has limited detection capabilities for weak or transient interactions; the Y2H system can identify both weak and transient interactions. However, it has high false-positive and false-negative rates. Protein Microarray technology offers a high degree of automation. Nevertheless, its application relies on the preparation of high-quality proteins, which leads to high costs. Protein Cross-Linking technology can provide structural information on protein binding sites with a relatively high detection throughput. However, this technique is complex and relies on the cross-linkability of specific amino acids. Despite these limitations, these experimental methods have accumulated a large amount of PPI data. Researchers have developed various databases, such as DIP [13], BioGRID [14], and STRING [15], to store, manage, and share these data. These databases provide a data foundation for the development of computational methods.

Computational methods for PPI prediction can be roughly classified into three types: sequence-based methods, structure-based methods, and network-based methods.

Sequence-based methods can be further divided into two types: protein sequence-based methods and gene sequence-based methods. Protein sequence-based methods mainly utilize amino acid composition, positional information, and the structural and functional information implicitly encoded in the sequence to construct computational methods. Typically, these methods extract various features from protein sequences, such as PSSM [16], conjoint triad [17], auto covariance [18], local descriptors [19], and pseudo amino acid composition [20], to characterize different protein attributes. Subsequently, based on the extracted features, machine learning or deep learning methods are employed to study PPIs. For example, Chen et al. [21] extracted protein sequence features, including pseudo amino acid composition, autocorrelation descriptors, local descriptors, and conjoint triad, and then utilized an elastic net for feature selection. This method employed LightGBM as a classifier that takes the optimized feature set as input to study PPIs. Zhang et al. [22] proposed a DeepSG2PPI model for PPI prediction. In addition, the methods in references [23,24,25,26,27] also extract features from protein sequences and then combine machine learning or deep learning methods to study PPIs.

Since protein sequences are encoded by gene sequences, some scholars study PPIs from the perspective of gene sequences. Zhou et al. [28] observed that the usage of codon pairs in interacting protein pairs is not random. Based on this discovery, they proposed using the frequency difference in codon pairs as input features to an SVM for identifying PPIs. Najafabadi et al. [29] utilized the relative frequency difference in codon pairs in interacting protein pairs as features and combined them with the naive Bayesian classifier to study PPIs. Zhao et al. [30] extracted three kinds of features from gene sequences (nucleotide features, dinucleotide features, and trinucleotide features) and combined them with an SVM to predict PPIs.

Structure-based methods mainly utilize the known three-dimensional structures of proteins to study PPIs. However, the number of protein three-dimensional structures determined experimentally is limited, which restricts the wide application of such methods. With the DeepMind team proposing AlphaFold [31], AlphaFold2 [32], AlphaFold3 [33], and its variant AlphaFold-Multimer [34], which can predict the three-dimensional structure of monomers and multi-body protein complexes with high accuracy, some researchers have begun to use predicted three-dimensional structures of proteins to study PPIs. Song et al. [35] utilized the three-dimensional structures of proteins predicted by AlphaFold2 to predict PPIs. This method employs the graph attention networks to extract spatial structure information of proteins and combines a 1D-CNN to extract sequence features. Then, it fuses the two types of features into a natural vector. Finally, the natural vectors of two proteins are input into a fully connected layer to predict the probability of interaction between them. Hu et al. [36] utilized image processing strategies to extract structural information from protein complexes predicted by AlphaFold-Multimer and combined deep neural networks to predict PPIs. Furthermore, Struct2Graph [37], FoldDock [38], SpeedPPI [39], SpatialPPIv2 [40], and PPI-BAN [41] methods are all based on protein structure information to study PPIs.

Network-based methods utilize the information between nodes in the PPI networks to study potential PPIs. Kovács et al. [42] proposed a method named L3, which posits that the more paths of length 3 between two protein nodes, the more likely they are to interact. Yuen et al. [43] revised the L3 principle from the perspective of network modeling and proposed the NormalizedL3 (L3N) method for predicting PPIs. Zeng et al. [44] developed the GNNGL-PPI method based on the graph isomorphism networks (GIN) to predict PPIs. Additionally, HIGH-PPI [45] and FFANE [46] methods are both based on PPI network information to study PPIs.

In this paper, we study PPIs based on protein sequences and gene sequences, combined with CNNs. First, we extracted three categories of features from protein sequence: global physicochemical properties features of the protein sequence, local same type of amino acid position variation features, and protein evolutionary conservation features, totaling 480 dimensions. Specifically, we used APAAC method to extract global hydrophobicity and hydrophilicity features of the protein sequence; we divided protein sequence into multiple continuous fragments, and converted each protein sequence fragment into digital sequences based on same type of amino acid interval distance sequence defined by us, then four basic statistics were extracted from each digital sequence to characterize the local same type of amino acid position variation features; we employed PSI-BLAST program (version 2.16.0) to generate a PSSM for the protein, and extracted its evolutionary conservation features from the PSSM through θ interval deviation product factor defined by us. Second, we extracted the frequency of single nucleotides, dinucleotides, trinucleotides, and the position features of single nucleotides, totaling 96 dimensions. For each protein, we extracted a total of 576 features and subsequently converted them into a 36 × 16 feature matrix for representation. Finally, we constructed a CNN model that takes the combined feature matrices of two proteins as input to predict the probability of interaction between them.

## 2. Materials and Methods

### 2.1. Datasets

We collected four PPI datasets from the Database of Interaction Proteins (DIP), including *Saccharomyces cerevisiae* (*S. cerevisiae*), *Drosophila melanogaster* (*D. melanogaster*), *Homo sapiens* (*H. sapiens*), and *Mus musculus* (*M. musculus*). The datasets were processed according to the following two conditions: proteins with sequence lengths less than 50 amino acids were removed; the CD-HIT [47] software (version 4.8.1) was employed to remove redundancy from the dataset, with the sequence similarity threshold set at 40%. After the above processing, *S. cerevisiae* dataset contains 13,134 interacting protein pairs, *D. melanogaster* dataset contains 11,234 interacting protein pairs, *H. sapiens* dataset contains 3385 interacting protein pairs, and *M. musculus* dataset contains 837 interacting protein pairs. The number of proteins included in each species is shown in Table S1.

Currently, DIP only contains interacting protein pairs (positive samples). In the study of PPIs, non-interacting protein pairs (negative samples) are usually constructed manually. Common methods for constructing negative samples include the k-lets strategy [17], the random strategy [20], and the subcellular localization strategy [48]. Previous studies have shown that proteins located in different subcellular compartments generally do not interact with each other [49]. Therefore, we used the subcellular localization strategy to construct negative samples. The specific process was as follows: first, the positive samples were randomly divided into a training set, a validation set, and a test set at a ratio of 3:1:1; second, negative samples corresponding to each set were constructed using the subcellular localization strategy. The number of positive and negative samples for each dataset across four species is shown in Table S1. Here, the subcellular localization information for all proteins was sourced from the UniProt database. During the construction of negative samples, proteins with missing or ambiguous localization annotations were directly excluded; further, for proteins with multiple localization annotations, we retained only those simultaneously located in both the nuclear and cytoplasmic compartments.

The gene sequence corresponding to each protein was all obtained from the National Center for Biotechnology Information (NCBI) database. These gene sequences are coding sequences (CDS).

### 2.2. Feature Extraction

#### 2.2.1. Protein Sequence Features

In this paper, we used Ω ($\Omega =\left[A,C,D,E,F,G,H,I,K,L,M,N,P,Q,R,S,T,V,W,Y\right]$) to represent the set composed of 20 types of amino acids. Therefore, a protein sequence P of length L is expressed using Formula (1).

(1) $$P=P_{1}P_{2}\cdots P_{i}\cdots P_{L}\;\;\left(P_{i}\in \Omega ,i=1,2,\ldots ,L\right)$$

The protein structure and function, as well as their interactions with other molecules, are closely related to the physicochemical properties of amino acids. References [50,51] studied PPIs using two physicochemical properties of amino acid hydrophobicity and hydrophilicity, and achieved good results. Therefore, we also used these two properties of amino acids to study PPIs. Table S2 shows the hydrophobicity and hydrophilicity values of each amino acid. Due to the presence of negative values in the hydrophobicity and hydrophilicity values of amino acids, we applied the min–max normalization method to scale these values to the range between 0 and 1, as shown in Formula (2).

(2) $$x^{*}=\frac{x-x_{min}}{x_{max}-x_{min}}$$

Based on the normalized hydrophobicity and hydrophilicity values of amino acids, the protein sequence P was mapped into 2 numerical sequences, as shown in Formula (3).

(3) $$P^{j}=\varphi_{1}^{j}\varphi_{2}^{j}\cdots \varphi_{L}^{j}\;\;\left(j=1,2\right)$$

where $\varphi_{i}^{1}$ represents the hydrophobicity value of the amino acid at the i-th position in the protein sequence, $\varphi_{i}^{2}$ represents the hydrophilicity value of the amino acid at the i-th position in the protein sequence.

We utilized the APAAC method to extract the global hydrophobicity and hydrophilicity features of the protein sequence. APAAC was proposed by Chou [52] and comprises three parts of information: amino acid composition $f_{m}$; hydrophobicity correlation factor $\tau_{2d-1}$; and hydrophilicity correlation factor $\tau_{2d}$. The specific calculation formulas are shown in Formulas (4)–(8). Among them, the hydrophobicity correlation factor $\tau_{2d-1}$ is used to calculate the average value of the product of the hydrophobicity values of all pairs of amino acids with an interval of d in the sequence, as shown in Formula (7); the hydrophilicity correlation factor $\tau_{2d}$ is used to calculate the average value of the product of the hydrophilicity values of all pairs of amino acids with an interval of d in the sequence, as shown in Formula (8).

(4) $$V=(v_{1},v_{2},\cdots ,v_{20},v_{20+1},v_{20+2},\cdots ,v_{20+2\lambda })$$

(5) $$v_{t}=\left\{\begin{array}{c} \frac{f_{m}}{1+\omega \sum_{k=1}^{2\lambda }\tau_{k}},\;\;\;\;\;\;\;\;\;\;\;\;\;\;\;\;\;\;\;\;\;\;1\leq t\leq 20\\ \frac{\omega \tau_{t}}{1+\omega \sum_{k=1}^{2\lambda }\tau_{k}},\;\;\;\;\;\;\;\;\;\;\;21\leq t\leq 20+2\lambda \end{array}\right.$$

(6) $$f_{m}=\frac{n_{m}}{L}\;\;\;\;(m=1,2,\ldots ,20)$$

(7) $$\tau_{2d-1}=\frac{1}{L-d}\sum_{i=1}^{L-d}\varphi_{i}^{1}\times \varphi_{i+d}^{1}$$

(8) $$\tau_{2d}=\frac{1}{L-d}\sum_{i=1}^{L-d}\varphi_{i}^{2}\times \varphi_{i+d}^{2}$$

where ω is the weight factor, m represents the amino acid type, and $n_{m}$ indicates the number of times amino acid m appears in the protein sequence.

Considering the issue of feature redundancy, we drew on the relevant experience in references [50,51], and set the parameters in APAAC as ω = 0.5 and λ = 30, extracting 80 features to characterize the global hydrophobicity and hydrophilicity features of the protein sequence. The vector comprising these 80 features is denoted by $V_{1}$, as shown in Formula (9).

(9) $$V_{1}=(v_{1},v_{2},\cdots ,v_{20},v_{21},v_{22},\cdots ,v_{80})$$

To extract the local sequence features of the protein, we followed the approach in reference [53] and divided each protein sequence P into four consecutive segments. The specific division method is shown in Formulas (10)–(14). Suppose that the length of protein sequence P is L, and let $l=\left\lfloor \frac{L}{4}\right\rfloor$. The division method of the four fragments is as follows: the first sequence fragment contains the amino acids from position 1 to position l, as shown in Formula (11); the second sequence fragment contains the amino acids from position l + 1 to position 2l, as shown in Formula (12); the third sequence fragment contains the amino acids from position 2l + 1 to position 3l, as shown in Formula (13); the fourth sequence fragment contains the amino acids from position 3l + 1 to position L, as shown in Formula (14).

(10) $$P=H_{1}H_{2}H_{3}H_{4}$$

(11) $$H_{1}=P_{1}P_{2}\cdots P_{l}$$

(12) $$H_{2}=P_{l+1}P_{l+2}\cdots P_{2l}$$

(13) $$H_{3}=P_{2l+1}P_{2l+2}\cdots P_{3l}$$

(14) $$H_{4}=P_{3l+1}P_{3l+2}\cdots P_{L}$$

The positional information of each amino acid in the protein sequence contains important information about the protein structure. Therefore, we considered using the same type of amino acid interval distance variation information to study PPIs. We defined the same type of amino acid interval distance sequence $g_{m}\left(\tau \right)$, as shown in Formula (15).

In a protein sequence segment of length s, if amino acid m occurs q times, and the occurrence positions are sequentially represented by $\alpha_{1},\alpha_{2},\cdots ,\alpha_{q}$.

(15) $$g_{m}\left(\tau \right)=\left\{\begin{array}{ccc} \alpha_{\tau +1}-\alpha_{\tau } & 1<\tau <q-1 & \left(q>1\right)\\ s-\alpha_{1} & & \left(q=1\right)\;\\ 0 & & \left(q=0\right)\; \end{array}\right.$$

Each value in the same type of amino acid interval distance sequence represents the positional interval between adjacent amino acids of the same type, effectively preserving the positional distribution information of amino acids in the protein sequence. Therefore, the same type of amino acid interval distance sequence can be used for PPI prediction.

We extracted four features from the same type of amino acid interval distance sequence $g_{m}\left(\tau \right)$ as follows:

(1). The mean $A_{m}$ of the same type of amino acid interval distance sequence $g_{m}\left(\tau \right)$ is shown in Formula (16).

(16) $$A_{m}=\left\{\begin{array}{cc} \frac{\sum_{\tau =1}^{q-1}g_{m}\left(\tau \right)}{\left|g_{m}\left(\tau \right)\right|} & \left(q>1\right)\\ g_{m}\left(\tau \right) & \left(q=1\right)\\ 0 & \left(q=0\right) \end{array}\right.$$

(2). The minimum $B_{m}$ of the same type of amino acid interval distance sequence $g_{m}\left(\tau \right)$ is shown in Formula (17).

(17) $$B_{m}=min\left(g_{m}\left(\tau \right)\right)$$

(3). The maximum $C_{m}$ of the same type of amino acid interval distances sequence $g_{m}\left(\tau \right)$ is shown in Formula (18).

(18) $$C_{m}=max\left(g_{m}\left(\tau \right)\right)$$

(4). The frequency $D_{m}$ of amino acid m is shown in Formula (19).

(19) $$D_{m}=\left\{\begin{array}{cc} \frac{\left|g_{m}\left(\tau \right)\right|+1}{s} & \left(q>1\right)\\ \frac{1}{s} & \left(q=1\right)\\ 0 & \left(q=0\right) \end{array}\right.$$

where $\left|g_{m}\left(\tau \right)\right|$ denotes the length of $g_{m}\left(\tau \right)$.

According to Formula (15), we mapped a protein sequence fragment into 20 interval distance sequences of amino acids of the same type. For each type of amino acid interval distance sequence, we calculated four features using Formulas (16)–(19). Therefore, a sequence segment could extract 4 × 20 = 80 features. Each protein sequence was divided into four consecutive segments, from which we extracted 4 × 80 = 320 features to characterize the local same type of amino acid position variation features. The vector comprising these 320 features is denoted by $V_{2}$, as shown in Formula (20).

(20) $$V_{2}=\left(A_{m,1},\cdots ,D_{m,1},A_{m,2},\cdots ,D_{m,2},A_{m,3},\cdots ,D_{m,3},A_{m,4},\cdots ,D_{m,4}\right)$$

Jones et al. first introduced the PSSM into protein structural studies, and it can effectively represent the evolutionary conservation information of the protein sequence. We used the PSI-BLAST program (version 2.16.0) against the NCBI non-redundant (NR) sequence database released on 7 February 2024 to compute the PSSM for each protein. The running parameters of PSI-BLAST were configured as follows: E-value was set to 0.001, and the number of iterations was set to 3. The PSSM corresponding to a protein sequence P of length L is expressed using Formula (21). To eliminate the impact of dimensional differences in the raw PSSM data, we normalized it using the Sigmoid function, as shown in Formula (22).

(21) $$PSSM=\left[\begin{array}{ccc} a_{1,1} & \cdots & a_{1,20}\\ \vdots & \ddots & \vdots \\ a_{L,1} & \cdots & a_{L,20} \end{array}\right]$$

(22) $$a_{i,w}^{*}=\frac{1}{1+e^{-a_{i,w}}}(i=1,2,\ldots ,L,w=1,2,\ldots ,20)$$

The amino acid at each position in protein sequences carries evolutionary information about the protein. Moreover, during evolution, the amino acid at each position does not evolve independently but exhibits coevolutionary phenomena with amino acids at other positions. To quantify the coevolution of amino acids at different positions, we defined a mathematical descriptor—θ interval deviation product factor $\delta_{i,w}^{\theta }$. The calculation is based on the PSSM of the protein: for two positions in the protein sequence separated by θ, the deviation of the score of the amino acid at each position from the mean of the corresponding column is calculated, and then the two deviation values are multiplied. The resulting product is the θ interval deviation product factor $\delta_{i,w}^{\theta }$, and the calculation formula is shown in Formula (23). This mathematical descriptor aims to quantify the tendency of two amino acids at different positions to evolve into the same type of amino acid. Furthermore, we calculated the average value of all θ interval deviation product factors in each protein sequence to characterize the overall evolutionary conservation feature of the protein sequence, as shown in Formula (24).

(23) $$\delta_{i,w}^{\theta }=\left(a_{i,w}-{\bar{a}}_{w}\right)\left(a_{i+\theta ,w}-{\bar{a}}_{w}\right)$$

(24) $$Z_{w}^{\theta }=\frac{1}{L-\theta }\sum_{i=1}^{L-\theta }\delta_{i,w}^{\theta }$$

where

(25) $${\bar{a}}_{w}=\frac{\sum_{i=1}^{L}a_{i,w}}{L}\;\;(w=1,2,\cdots ,20)$$

Considering the issue of feature redundancy, we set the parameter θ to 1, 2, and 3. Based on this, we extracted 60 features of protein evolutionary conservation and represented these features by $\phi_{w}^{1}$, $\phi_{w}^{2}$, and $\phi_{w}^{3}\left(w=1,2,\cdots ,20\right)$. Meanwhile, we also took the average value of each column of the PSSM, denoted as ${\bar{a}}_{w}$, as a feature to describe protein evolutionary conservation, totaling 20 dimensions. Therefore, we extracted 80 features to characterize the evolutionary conservation of proteins. The vector comprising these 80 features is denoted by $V_{3}$, as shown in Formula (26).

(26) $$V_{3}=\left(\phi_{1}^{1},\phi_{2}^{1},\cdots \phi_{20}^{1},\phi_{1}^{2},\phi_{2}^{2},\cdots \phi_{20}^{2},\phi_{1}^{3},\phi_{2}^{3},\cdots \phi_{20}^{3},{\bar{a}}_{1},{\bar{a}}_{2},\cdots ,{\bar{a}}_{20}\right)$$

#### 2.2.2. Gene Sequence Features

We used E ($E=\left[A,C,G,T\right]$) to represent the set composed of 4 kinds of nucleotides. Therefore, a gene sequence S of length N is expressed using Formula (27).

(27) $$S=e_{1}e_{2}\cdots {e_{r}\cdots e}_{N}\;(e_{r}\in E,r=1,2,3,\cdots ,N)$$

We calculated the frequency of single nucleotides, dinucleotides, and trinucleotides in each gene sequence, totaling 84 features. Then we introduced nucleotide positional information and calculated the mean $\mu_{e}$ and variance $\sigma_{e}^{2}$ of the positions where nucleotide e appears in the gene sequence, as shown in Formulas (28)–(29). There are 4 types of nucleotides, thus obtaining 8 position-related features.

For any nucleotide e∈E, suppose that e occurs ξ times in a gene sequence of length N, and the occurrence positions are sequentially represented by $\beta_{1},\beta_{2},\cdots ,\beta_{\xi }$.

(28) $$\mu_{e}=\frac{1}{\xi }\sum_{z=1}^{\xi }\beta_{z}$$

(29) $$\sigma_{e}^{2}=\frac{1}{\xi }\sum_{z=1}^{\xi }(\beta_{z}-\mu_{e}{)}^{2}$$

We also defined a mapping function $S_{r}$ to map all nucleotides in the gene sequence onto a unit circle, as shown in Formula (30). Under this mapping rule, nucleotide A in the gene sequence S is mapped to the first quadrant, nucleotide C to the second quadrant, nucleotide G to the third quadrant, and nucleotide T to the fourth quadrant. The coordinates of the nucleotide $e_{r}$ at the r-th position in the gene sequence S after mapping to the unit circle are given by ($x_{r},y_{r}$). The mapping function $S_{r}$ not only utilizes the type of nucleotide but also combines the position information of each nucleotide, as well as the number of that type of nucleotide up to that position in the sequence. Therefore, this representation method preserves the important information of the gene sequence S, enabling its application to predict PPIs.

(30) $$S_{r}=\left\{\begin{array}{c} \cos\left(\frac{\pi }{2}\times \frac{b_{A_{r}}}{b_{A}+1}\right),\;\sin\left(\frac{\pi }{2}\times \frac{b_{A_{r}}}{b_{A}+1}\right)\\ \cos\left(\frac{\pi }{2}+\frac{\pi }{2}\times \frac{b_{C_{r}}}{b_{C}+1}\right),\sin\left(\frac{\pi }{2}+\frac{\pi }{2}\times \frac{b_{C_{r}}}{b_{C}+1}\right)\\ \cos\left(\pi +\frac{\pi }{2}\times \frac{b_{G_{r}}}{b_{G}+1}\right),\sin\left(\pi +\frac{\pi }{2}\times \frac{b_{G_{r}}}{b_{G}+1}\right)\\ \cos\left(\frac{3\pi }{2}+\frac{\pi }{2}\times \frac{b_{T_{r}}}{b_{T}+1}\right),\sin\left(\frac{3\pi }{2}+\frac{\pi }{2}\times \frac{b_{T_{r}}}{b_{T}+1}\right)\;\; \end{array}\right.\;\;\;\;r=1,2,3,\cdots ,N$$

where $b_{e}$ (e=A,C,G,T) denotes the total number of nucleotide e in the gene sequence, and $b_{e_{r}}$ denotes the cumulative count of nucleotide e within the first r positions of the gene sequence.

**Figure 1:**
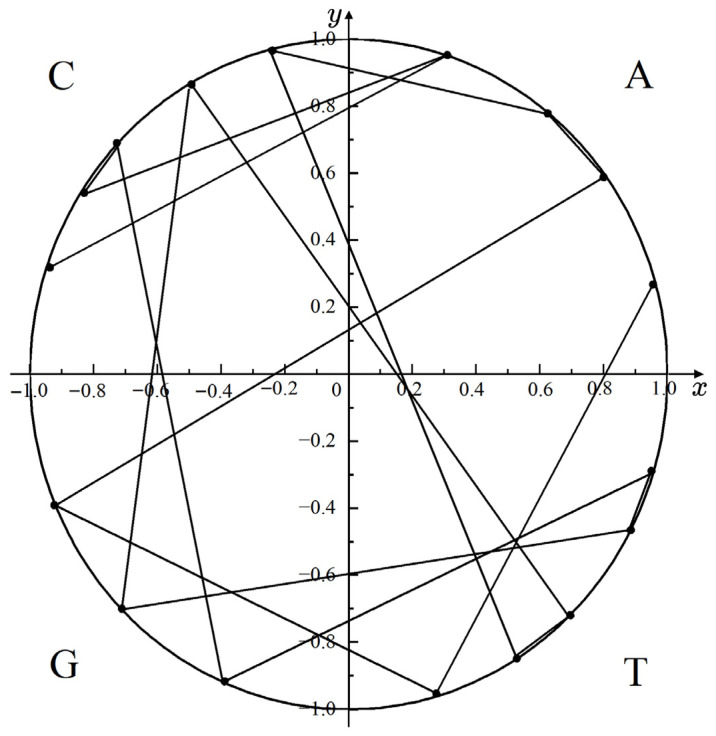
The mapping result of the gene sequence fragment ATGAACTTCGTTGCCAC.

We presented the mapping result of the gene sequence fragment ATGAACTTCGTTGCCAC onto the unit circle (see Figure 1).

To quantify the information contained in the mapped points, we extracted four features from the mapped points: the mean of the x-coordinates of all points, denoted as $\mu_{x}$, as shown in Formula (31); the mean of the y-coordinates of all points, denoted as $\mu_{y}$, as shown in Formula (32); the variance of the x-coordinates of all points, denoted as $\sigma_{x}^{2}$, as shown in Formula (33); the variance of the y-coordinates of all points, denoted as $\sigma_{y}^{2}$, as shown in Formula (34). Among them, the $\mu_{x}$ and $\mu_{y}$ reflect the relative number of nucleotides A,C,G, and T in the gene sequence. Specifically, when $\mu_{x}$ > 0, it indicates that the sum of A and T in the gene sequence is greater than that of G and C; conversely, when $\mu_{x}$ < 0, it indicates that the sum of A and T is less than that of G and C. When $\mu_{y}$ > 0, it indicates that the sum of A and C is greater than that of G and T; conversely, when $\mu_{y}$ < 0, it indicates that the sum of A and C is less than that of G and T.

(31) $$\mu_{x}=\frac{1}{N}\sum_{r=1}^{N}x_{r}$$

(32) $$\mu_{y}=\frac{1}{N}\sum_{r=1}^{N}y_{r}$$

(33) $$\sigma_{x}^{2}=\frac{1}{N}\sum_{r=1}^{N}{\left(x_{r}-\mu_{x}\right)}^{2}$$

(34) $$\sigma_{y}^{2}=\frac{1}{N}\sum_{r=1}^{N}{\left(y_{r}-\mu_{y}\right)}^{2}$$

Thus, we extracted 96 features from each gene sequence. The vector comprising these features is denoted by $V_{4}$, as shown in Formula (35).

(35) $$V_{4}=\left(\mu_{x},\mu_{y},\sigma_{x}^{2},\sigma_{y}^{2},\mu_{A},\cdots ,\mu_{T},\sigma_{A}^{2},\cdots ,\sigma_{T}^{2},f_{A},\cdots ,f_{TTT}\right)$$

### 2.3. Constructing Sample Feature Grayscale Maps

As described in Section 2.2 on feature extraction, for each protein, we extracted 576 features from the protein sequence and gene sequence. We transformed 576 features into a 36 × 16 feature matrix (see Formula (36)). The feature matrices corresponding to two proteins were then concatenated to form a 36 × 32 feature matrix, which is used to represent the protein pair sample generated by these two proteins.

**Figure 2:**
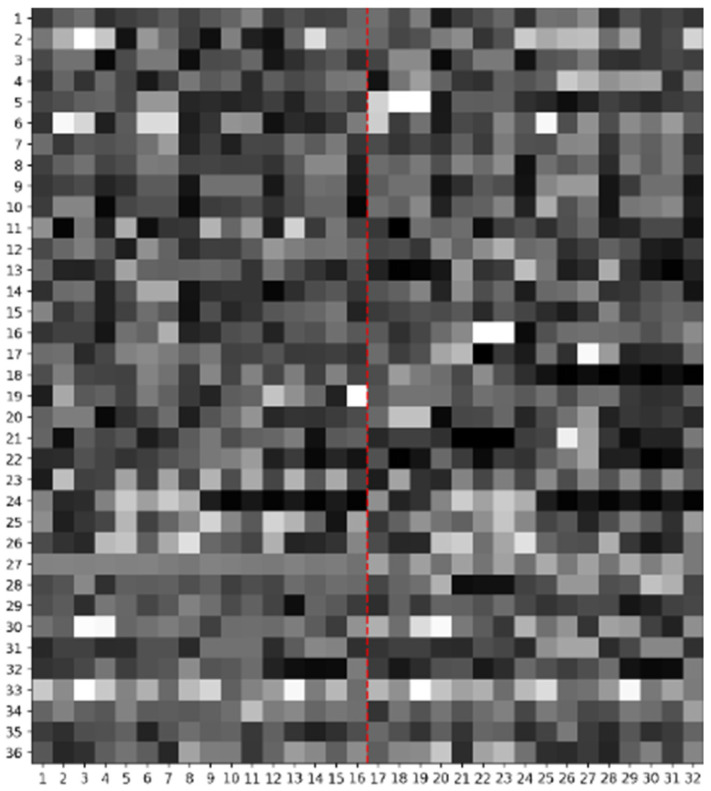
A sample feature grayscale map.

Due to the different scales of each feature, we applied the min–max normalization method to normalize the feature matrix of each sample, scaling all values to the range between 0 and 1. The specific min–max normalization formula is shown in Formula (2). After normalization, we regarded this feature matrix as a sample feature grayscale map, where the value of each element corresponds to the pixel brightness at the corresponding position in the image, with larger values making the pixel brighter and smaller values making the pixel darker. Figure 2 shows a sample feature grayscale map.

(36) $$\left[\begin{array}{ccccc} A_{1,1} & B_{1,1} & \cdots & C_{1,4} & D_{1,4}\\ \vdots & \vdots & \ddots & \vdots & \vdots \\ A_{20,1} & B_{20,1} & \cdots & C_{20,4} & D_{20,4}\\ v_{1} & v_{2} & \cdots & v_{15} & v_{16}\\ \vdots & \vdots & \ddots & \vdots & \vdots \\ v_{65} & v_{66} & \cdots & v_{79} & v_{80}\\ {\bar{a}}_{1} & {\bar{a}}_{2} & \cdots & {\bar{a}}_{15} & {\bar{a}}_{16}\\ \vdots & \vdots & \ddots & \vdots & \vdots \\ \phi_{5}^{3} & \phi_{6}^{3} & \cdots & \phi_{19}^{3} & \phi_{20}^{3}\\ \mu_{x} & \mu_{y} & \cdots & f_{G} & f_{T}\\ \vdots & \vdots & \ddots & \vdots & \vdots \\ f_{TAA} & f_{TAC} & \cdots & f_{TTG} & f_{TTT} \end{array}\right]$$

### 2.4. Constructing CNN Model

**Figure 3:**
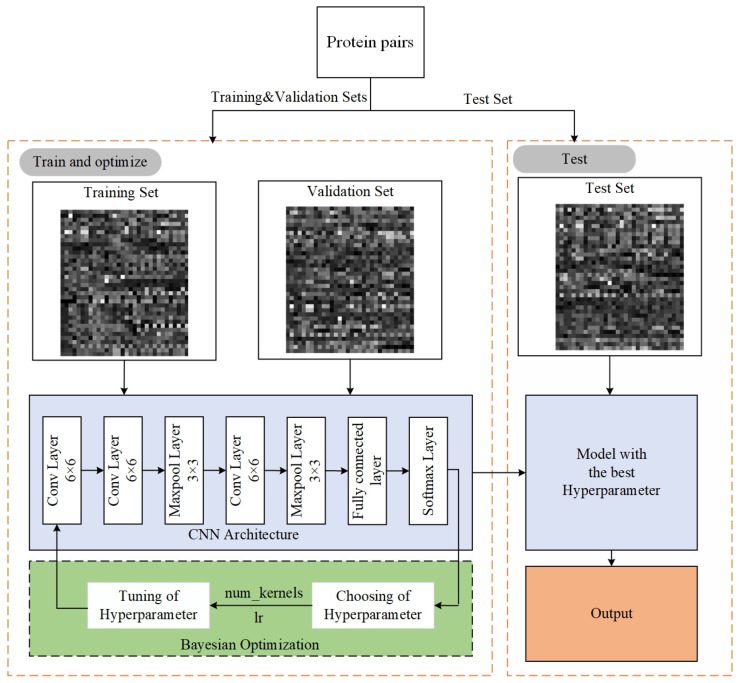
Our model’s architecture diagram.

**Figure 4:**
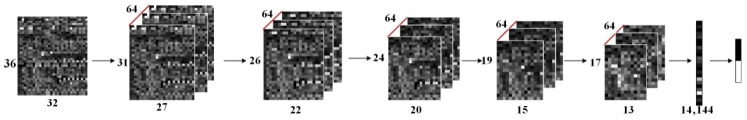
The various transformations of a sample feature grayscale map in the CNN model.

CNNs are feedforward neural networks that can automatically extract high-dimensional features from input images through convolutional layers and pooling layers, achieving accurate predictions of targets. We developed a CNN model using the Python programming language and the PyTorch framework, as illustrated in Figure 3. It consists of three convolutional layers, two pooling layers, and a fully connected layer. The three convolutional layers all employ 6 × 6 convolution kernels with 64 channels and a stride of 1 to extract spatial local features from the sample feature grayscale map. Each convolutional layer is followed by a ReLU activation function for a nonlinear transformation. The two pooling layers both use 3 × 3 max-pooling kernels with 64 channels and a stride of 1 to reduce the spatial dimensions of the sample feature grayscale map and enhance the translation invariance of the model. Figure 4 shows the transformation process of a sample feature grayscale map after being input into the CNN model.

After a sample feature grayscale map is input into the CNN model, it first passes through the first convolutional layer to generate 64 [31 × 27] matrices. Then, through the second convolutional layer, 64 [26 × 22] matrices are generated. These matrices are further processed through the first pooling layer to produce 64 [24 × 20] matrices. Subsequently, these matrices pass through the third convolutional layer, generating 64 [19 × 15] matrices. After that, these matrices are processed by the second pooling layer to produce 64 [17 × 13] matrices. Finally, the 64 [17 × 13] matrices are flattened into a [1 × 14,144] vector, and processed through a fully connected layer and a Softmax layer to output a [1 × 2] vector, where the two elements represent the probabilities of interaction and non-interaction between the two proteins in the sample, respectively.

The CNN models have various hyperparameters, which significantly impact their performance. Commonly used hyperparameter optimization methods include grid search, random search, and Bayesian optimization. The grid search method exhaustively explores all possible hyperparameter combinations within a predefined search space. Although it can find the optimal solution, its efficiency is comparatively low. Therefore, we employed the TPE algorithm to optimize two key hyperparameters in the CNN model: learning rate and number of convolutional kernels. The search space for the learning rate ranged from [0.00001, 0.001], while the number of convolutional kernels was chosen from the set [$2^{1},2^{2},\cdots ,2^{6}$]. We set the maximum number of trials for TPE hyperparameter optimization to 3, with the objective of maximizing the AUC value of the validation set. To prevent overfitting, we implemented an early stopping mechanism: if the validation loss does not improve for 10 epochs, the current experiment is terminated. In addition, the other parameters in the model were set as follows: a batch size of 128, an epoch of 100, the cross-entropy loss function was used to calculate the average loss of each batch of samples, and the Adam optimizer was used to optimize the network weights and bias parameters.

### 2.5. Evaluation Criteria

We used six evaluation indicators to evaluate model performance, specifically sensitivity, specificity, F1-score, MCC, accuracy, and AUC. The formulas for these evaluation indicators are defined in Formulas (37)–(42).

(37) $$sensitivity=\frac{TP}{TP+FN}$$

(38) $$specificity=\frac{TN}{TN+FP}$$

(39) $$precision=\frac{TP}{TP+FP}$$

(40) $$F1\text{-}score=\frac{2\times precision\times sensitivity}{precision+sensitivity}$$

(41) $$MCC=\frac{TP\times TN-FP\times FN}{\sqrt{\left(TP+FN\right)\left(TN+FP\right)\left(TP+FP\right)\left(TN+FN\right)}}$$

(42) $$accuracy=\frac{TP+TN}{TP+TN+FP+FN}$$

## 3. Results

### 3.1. Experimental Process

**Figure 5:**
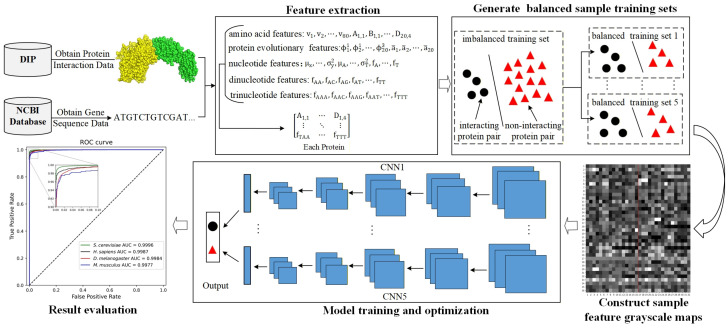
The specific experimental workflow.

The experimental process can be divided into five steps, as illustrated in the flowchart shown in Figure 5.

The first step was feature extraction. We extracted 576 features from each protein. The specific extraction process is detailed in Section 2.2.

The second step was to generate balanced sample training sets. We employed the subcellular localization strategy to construct negative samples for four species datasets. Since the number of generated negative samples significantly exceeds that of positive samples, directly training the model with this data would cause the trained model to be biased towards the majority class. Therefore, we applied an undersampling strategy to construct balanced sample training sets for each dataset. For each dataset, first, we randomly sampled five times from the negative samples of the training set to generate five negative sample sets, with each negative sample set having the same number of samples as the positive samples in that training set. Second, each sampled negative sample set was combined with all positive samples to form balanced sample training sets. Finally, each dataset yielded five balanced sample training sets.

The third step was to construct sample feature grayscale maps. According to Section 2.3, we constructed sample feature grayscale maps for all samples in the four datasets.

The fourth step was model training and optimization. We trained CNN models using five balanced sample training sets of each dataset and obtained five independent CNN models named CNN1, …, CNN5. Subsequently, we tuned the hyperparameters for each CNN model using the validation set. The optimal hyperparameter configurations of the five independent CNN models on each dataset (*S. cerevisiae*, *D. melanogaster*, *H. sapiens*, and *M. musculus*) are provided in Tables S3–S6. Finally, we took the average of the output results from the five models as the final prediction. Experiments were conducted on the Linux system with 125GB of RAM and NVIDIA GeForce RTX 3080Ti GPU. The training times for the four datasets were approximately 2.3 h for *S. cerevisiae*, 2.8 h for *D. melanogaster*, 1.5 h for *H. sapiens*, and 0.3 h for *M. musculus*.

The fifth step was result evaluation. Similarly, due to the severe imbalance between positive and negative samples in the test set, we applied the same generation method as for the balanced sample training set to construct a balanced sample test set. We utilized the CNN model with the optimal hyperparameter configuration to predict the balanced sample test set and evaluated the predictions.

### 3.2. Analysis of Test Set Results

We evaluated the predictions of our method on the test sets of four species using five evaluation indicators. As shown in Table 1, on the four test sets, the average sensitivity of our method was 97.29%, the average specificity was 99.15%, the average F1-score was 98.20%, the average MCC was 96.52%, and the average accuracy was 98.22%. This indicates that our method demonstrates high and stable predictive performance on the test sets. Specifically, this method achieved the highest classification performance on the *S. cerevisiae* dataset, with the following metrics and their corresponding standard deviations: sensitivity 99.19% (0.62%), specificity 99.38% (0.47%), F1-score 99.28% (0.27%), MCC 98.58% (0.54%), and accuracy 99.28% (0.27%). On the *D. melanogaster* and *H. sapiens* datasets, our method also demonstrated excellent predictive performance with sensitivities of 97.69% (1.49%) and 98.52% (0.81%), specificities of 98.61% (1.70%) and 98.72% (0.79%), F1-scores of 98.15% (0.95%) and 98.62% (0.30%), MCCs of 96.34% (1.90%) and 97.26% (0.60%), and accuracies of 98.15% (0.95%) and 98.62% (0.30%), respectively. On the *M. musculus* dataset, our method achieved a specificity of 99.87% (0.26%), indicating that the model could accurately identify the negative samples in this dataset. Its sensitivity was 93.77% (2.60%), suggesting that the model also had a relatively high recognition ability for the positive samples in this dataset, but it was slightly lower than that in identifying negative samples. Notably, the specificity of our method on the four test sets was above 98.5%, indicating that this method could effectively avoid misclassifying negative samples as positive samples. Overall, our method demonstrates high performance on multiple species test sets. Here, the numbers in parentheses represent the standard deviation calculated based on five predictions.

**Figure 6:**
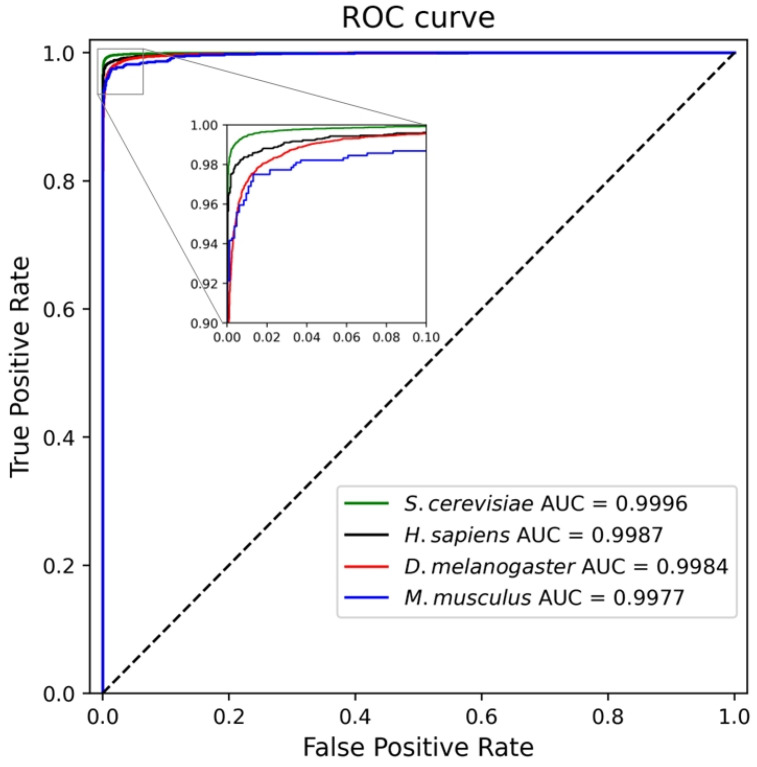
ROC curve of our method on four species test sets.

To further evaluate the classification performance of the model, we plotted its ROC curves on the test sets of four species (see Figure 6). In Figure 6, the green, black, red, and blue lines represent the results of the test sets of *S. cerevisiae*, *H. sapiens*, *D. melanogaster*, and *M. musculus*, respectively. As shown in Figure 6, our method achieved an AUC of 0.9996 on the *S. cerevisiae* test set, 0.9987 on the *H. sapiens* test set, 0.9984 on the *D. melanogaster* test set, and 0.9977 on the *M. musculus* test set, demonstrating excellent classification performance across four test sets.

**Figure 7:**
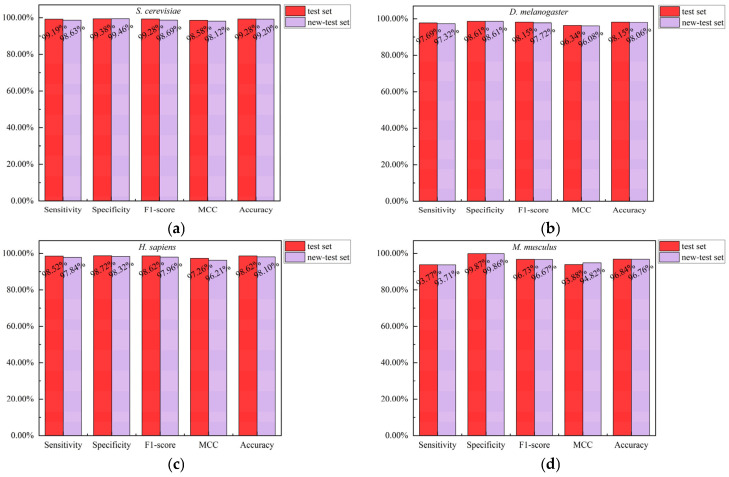
Comparison of predictions by the CNN model on the test set and the new-test set across four species. (**a**) *S. cerevisiae*; (**b**) *D. melanogaster*; (**c**) *H. sapiens*; (**d**) *M. musculus*.

To evaluate the robustness of the model, we ensured that the test set and training set were disjoint at the protein level. Specifically, we removed all interacting protein pairs from the test set that contained proteins present in the training set, thereby constructing a new test set, referred to as the new-test set. We used the optimal CNN model to predict the new-test set and compared the results with those of the test set (see Figure 7). As shown in Figure 7, the predictions of the CNN model were highly consistent across the two test sets for the four species. This indicates that the overlap of proteins between the test set and the training set has minimal impact on the model’s predictive performance.

We further analyzed the performance of the model on imbalanced datasets. We constructed two imbalanced test sets with positive-to-negative sample ratios of 1:5 and 1:10, referred to as test set 1 and test set 2, respectively. In test set 2, the negative samples from the *M. musculus* species accounted for 90.32% of all its negative samples. The predictions of the CNN model on the imbalanced test sets (test set 1 and test set 2) for four species are provided in Tables S7 and S8.

**Figure 8:**
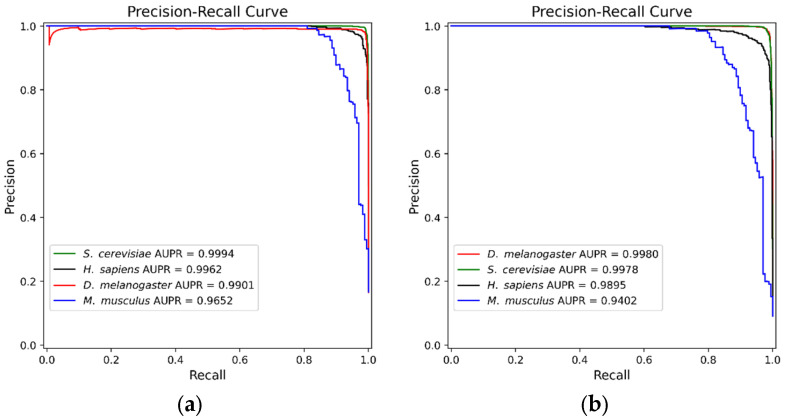
PR curves of the CNN model on imbalanced test sets for the four species. (**a**) PR curves on test set 1; (**b**) PR curves on test set 2.

We plotted the precision–recall (PR) curves for the predictions of four species on test set 1 and test set 2 (see Figure 8). In Figure 8, the green, black, red, and blue curves represent the predictions for the *S. cerevisiae*, *H. sapiens*, *D. melanogaster*, and *M. musculus*, respectively. As shown in Figure 8a, on test set 1, the CNN model achieved AUPR values of 0.9994, 0.9962, 0.9901, and 0.9652 for the *S. cerevisiae*, *H. sapiens*, *D. melanogaster*, and *M. musculus*, respectively. As shown in Figure 8b, on test set 2, the CNN model achieved AUPR values of 0.9978, 0.9895, 0.9980, and 0.9402 for the *S. cerevisiae*, *H. sapiens*, *D. melanogaster*, and *M. musculus*, respectively. The CNN model achieved high AUPR on two imbalanced test sets of four species, indicating its effectiveness in accurately identifying positive samples.

We presented the precision of the top u (u = 100, 200, and 300) predictions of the CNN model on test set 1 and test set 2 (see Table 2). As shown in Table 2, the CNN model demonstrated relatively stable performance on the *S. cerevisiae* and *H. sapiens* datasets. On test set 1 and test set 2, it achieved 100% precision for the top 100, 200, and 300 predictions. On the *D. melanogaster* dataset, the precision of the CNN model on test set 1 was slightly lower than that on test set 2, with a decrease of no more than 1%. On the *D. melanogaster* dataset, the precision on both test sets was greater than or equal to 99%. On the *M. musculus* dataset, the performance of the CNN model showed a relatively large fluctuation on test set 2. The precision for the top 100 predictions was 100%, while it dropped to 88.48% for the top 200 and top 300 predictions.

**Figure 9:**
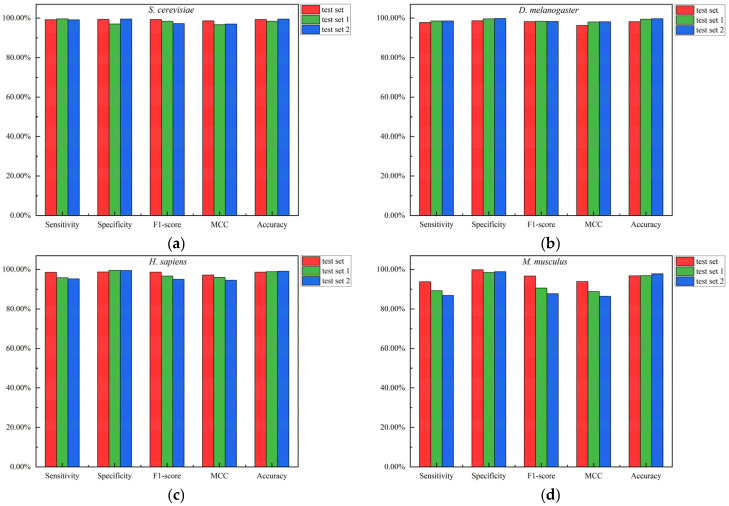
Comparison of the predictive performance of the CNN model on three test sets across four species. (**a**) *S. cerevisiae*; (**b**) *D. melanogaster*; (**c**) *H. sapiens*; (**d**) *M. musculus*.

We also compared the predictions of the CNN model on the balanced test set and two imbalanced test sets (test set 1 and test set 2) for the four species (see Figure 9). As shown in Figure 9, the predictive performance of the CNN model on the *S. cerevisiae* and *H. sapiens* species remained relatively stable, with nearly equivalent performance between the balanced and imbalanced test sets. In the *D. melanogaster* species, the performance of the CNN model on the imbalanced test sets slightly improved compared to the balanced test set, and the predictive performance was almost identical across the two imbalanced test sets. In the *M. musculus* species, the performance of the CNN model exhibited relatively larger fluctuations across the three test sets, with the F1-score showing the largest variation: 96.73% on test set, 90.63% on test set 1, and 87.68% on test set 2. The sensitivity, specificity, and MCC indicators also declined on the imbalanced test sets, the extent of decrease was relatively minor, while the accuracy indicator showed an increase. Based on the above analysis, the CNN model demonstrates relatively consistent predictive performance on both the balanced test set and the two imbalanced test sets.

### 3.3. Comparative Analysis with Existing Methods

On the *S. cerevisiae* dataset, we compared our method with eight methods: AE-LGBM [24], OLPP-ROF [26], LPBERT [27], SVM-NVDT [30], TAGPPI [35], PPI-BAN [41], SVM-FFANE [46], and CF-PPI [54] (see Table 3). The nine methods listed in Table 3 all used the same positive sample dataset and constructed negative samples using the subcellular localization strategy. For test set construction, the nine methods remained consistent, constructing balanced test sets with a 1:1 ratio of positive to negative samples. The results listed in Table 3 represent the predictive performance of each method on its corresponding test set.

As shown in Table 3, our method and the SVM-NVDT method significantly outperformed the AE-LGBM, OLPP-ROF, LPBERT, TAGPPI, PPI-BAN, SVM-FFANE, and CF-PPI methods in sensitivity, F1-score, and accuracy, with our method being slightly superior to the SVM-NVDT method in these three indicators. In specificity, our method achieved 99.38%, outperforming AE-LGBM (98.70%), LPBERT (98.67%), TAGPPI (98.10%), PPI-BAN (98.15%), and CF-PPI (96.12%). In MCC, our method achieved the highest value of 98.58%, significantly outperforming the other eight methods, followed by the TAGPPI method at 97.80%, while the OLPP-ROF method had the lowest MCC at 82.10%.

On the *H. sapiens* dataset, we compared our method with eight methods: OLPP-ROF [26], LPBERT [27], SVM-NVDT [30], SVM-FFANE [46], ESMDNN-PPI [55], DeepTrio [56], RF-LPQ [57], and DeepInteract [58] (see Table 4). The nine methods listed in Table 4 all used the same positive sample dataset and constructed negative samples using the subcellular localization strategy. For test set construction, the nine methods remained consistent, constructing balanced test sets with a 1:1 ratio of positive to negative samples. The results listed in Table 4 represent the predictive performance of each method on its corresponding test set.

As shown in Table 4, our method significantly outperformed the OLPP-ROF, LPBERT, SVM-NVDT, SVM-FFANE, ESMDNN-PPI, DeepTrio, RF-LPQ, and DeepInteract methods in both MCC and accuracy. In sensitivity, our method achieved 98.52%, performing better than six other methods: OLPP-ROF (95.20%), SVM-FFANE (96.95%), ESMDNN-PPI (84.49%), DeepTrio (97.23%), RF-LPQ (97.32%), and DeepInteract (90.47%), though it was lower than SVM-NVDT (99.90%). In specificity, our method achieved 98.72%, which was higher than the LPBERT (98.58%) and DeepInteract (98.47%) methods, but was lower than the ESMDNN-PPI and DeepTrio methods. In F1-score, our method achieved 98.62%, outperforming SVM-NVDT (95.61%), SVM-FFANE (98.00%), and DeepTrio (98.11%).

### 3.4. Application of Our Method to PPI Networks Prediction

We extended our method to the prediction of PPI networks. We collected partial PPI network data of *S. cerevisiae* from the STRING database by setting the interaction confidence score threshold to 0.9, obtaining 401 interacting protein pairs. This dataset involves 51 proteins. The 401 interacting protein pairs show no overlap with the 13,134 interacting protein pairs recorded in the DIP. However, among the 51 proteins that constitute these interacting protein pairs, 31 proteins are present in the DIP.

**Figure 10:**
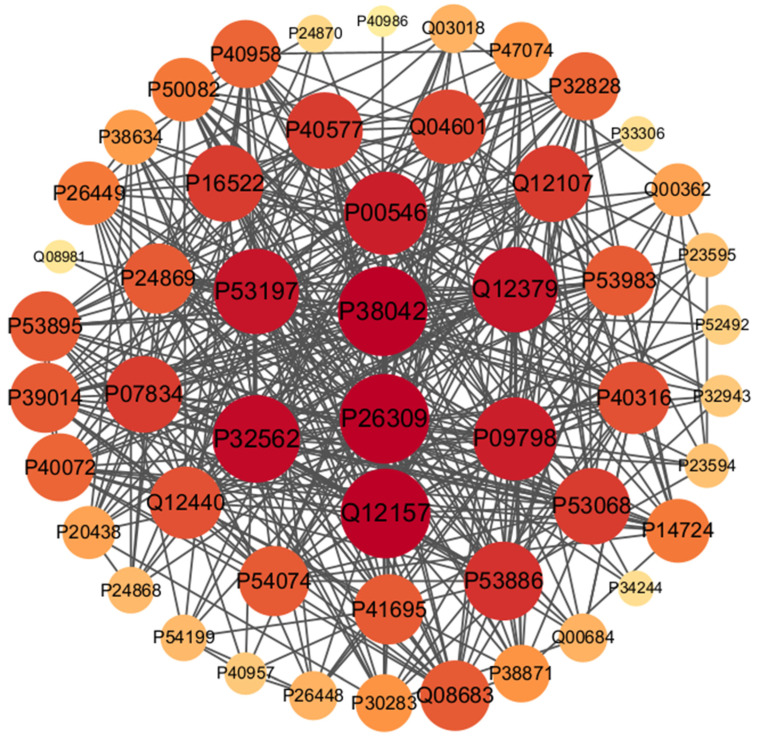
The predictions of the PPI network dataset.

We utilized the model trained and optimized on the *S. cerevisiae* dataset to make predictions on the PPI network dataset, and set five classification thresholds (0.5, 0.8, 0.9, 0.95, and 0.98) to evaluate the predictions (see Table 5). As shown in Table 5, when the classification threshold was set to 0.98 (i.e., samples with a predicted probability value greater than 0.98 are classified as interacting protein pairs), the sensitivity, precision, F1-score, and accuracy of our method on the PPI network dataset were all 100%. We used the Cytoscape software (version 3.10.0) [59] to visualize the predictions, as shown in Figure 10. In Figure 10, we use nodes to represent proteins, where higher-degree proteins are represented by larger and darker nodes, while lower-degree proteins are represented by smaller and lighter nodes. The correctly predicted interacting protein pairs are connected by black lines. As can be seen from Figure 10, our method correctly predicted all the interacting protein pairs in this network.

We further extended our method to the prediction of protein–protein non-interaction (PPNI) networks. We collected three types of PPNI network datasets from the Negatome database, including a one-core network dataset, a multiple-core network dataset, and a crossing network dataset. The one-core network is characterized by the presence of a core protein that exhibits no interactions with others in the network. The multiple-core network is composed of several one-core networks, with no interactions among the core proteins of these different one-core networks. The crossing network comprises both one-core networks and multiple-core networks. In this paper, the one-core network dataset contains 27 proteins, where P62879 is the core protein. The multiple-core network dataset contains 26 proteins, including 82 non-interacting protein pairs. The crossing network dataset involves 73 *H. sapiens* proteins, with 81 non-interacting protein pairs. For the three types of *H. sapiens* PPNI network datasets collected from the Negatome database, there is partial overlap in the protein composition with the *H. sapiens* proteins recorded in the DIP: 8 out of 27 *H. sapiens* proteins in the one-core network dataset are repeated, 9 out of 26 *H. sapiens* proteins in the multiple-core network dataset are repeated, and 46 out of 73 *H. sapiens* proteins in the crossing network dataset are repeated.

We applied our method to three PPNI network datasets and evaluated the predictions using five classification thresholds (0.5, 0.2, 0.1, 0.05, and 0.01), as shown in Table 6. When the classification threshold was set to 0.01 (i.e., samples with a predicted probability value less than 0.01 are classified as non-interacting protein pairs), the specificity and accuracy of our method on the three PPNI network datasets were all 100%. This result indicates that our method can be effectively transferred from the task of predicting interacting protein pairs to that of predicting non-interacting protein pairs.

### 3.5. Analysis of the Feature and Strategies’ Importance

**Figure 11:**
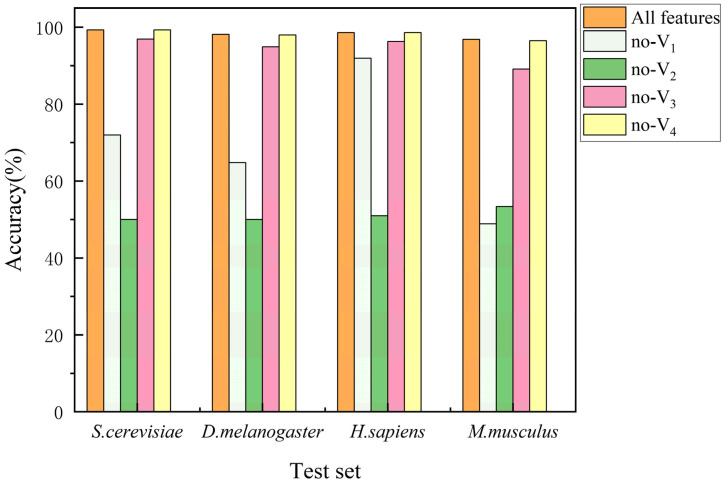
The impact of different feature types on model performance.

To evaluate the impact of features on model performance, we classified extracted features into four categories: global physicochemical properties features of the protein sequence $V_{1}$, local same type of amino acid position variation features $V_{2}$, protein evolutionary conservation features $V_{3}$, and gene sequence features $V_{4}$. Then we implemented a feature ablation strategy that sequentially sets each type of feature value to zero, assessing the contribution of different feature types to model performance by observing changes in accuracy (see Figure 11).

When four types of features were sequentially set to zero, the accuracy declined to varying degrees, indicating that all four feature types contributed to model performance. Among them, when local same type of amino acid position variation features were set to zero, the accuracy dropped most significantly. The accuracy on the test sets of *S. cerevisiae*, *D. melanogaster*, *H. sapiens*, and *M. musculus* dropped from 99.28%, 98.15%, 98.62%, and 96.84% to 50%, 50%, 51%, and 53.46%, respectively, representing a decline of nearly half. The next was the global physicochemical properties features of the protein sequence. When global physicochemical properties features of the protein sequence were set to zero, the accuracy on *M. musculus* test set dropped from 96.84% to 48.87%, a decrease of approximately half; on *D. melanogaster* test set, the accuracy dropped from 98.15% to 64.77%, a decrease of one-third; on *S. cerevisiae* test set, the accuracy dropped from 99.28% to 71.96%, a decrease of one-fourth; on *H. sapiens* test set, the impact was relatively small, with the accuracy dropping from 98.62% to 91.94%, a decrease of 6.68%. The protein evolutionary conservation features and gene sequence features also influenced model performance, but their impact was considerably smaller compared to global physicochemical properties features of the protein sequence and local same type of amino acid position variation features. Based on the above analysis, it can be concluded that among the four categories of features, the local same type of amino acid position variation features play a dominant role in the prediction process and have the most significant impact on the performance of the CNN model.

The dimension of the standard PSSM is L × 20 (L is the length of the protein sequence). Due to the varying lengths of protein sequences, directly using the PSSM as the protein evolutionary conservation features would lead to inconsistent feature dimensions. Therefore, the column mean ${\bar{a}}_{w}$ of the PSSM is often used to characterize protein evolutionary conservation features. To evaluate the effectiveness of our proposed $Z_{w}^{\theta }$ features and the $V_{3}$ features, we conducted two additional experiments: Experiment 1, using only the ${\bar{a}}_{w}$ features as the protein evolutionary conservation features, with all other features unchanged; and Experiment 2, using only the $Z_{w}^{\theta }$ features as the protein evolutionary conservation features, with all other features unchanged. The results of these two experiments were compared with those of the Experiment using the $V_{3}$ features as the protein evolutionary conservation features (see Table 7).

As shown in Table 7, in the *S. cerevisiae* species, the accuracy of Experiment 1 was slightly higher than that of Experiment 2; however, in the three species of *D. melanogaster*, *H. sapiens*, and *M. musculus*, the accuracy of Experiment 2 was consistently better than that of Experiment 1. Nevertheless, across all four species, the accuracy of both Experiment 1 and Experiment 2 was lower than that of the experimental results using the $V_{3}$ features. The above analysis indicates that the impact of our defined $Z_{w}^{\theta }$ features on model performance is generally superior to that of the standard ${\bar{a}}_{w}$ features, and that the $V_{3}$ features we proposed have a superior impact on model performance compared to both the $Z_{w}^{\theta }$ and ${\bar{a}}_{w}$ features.

**Figure 12:**
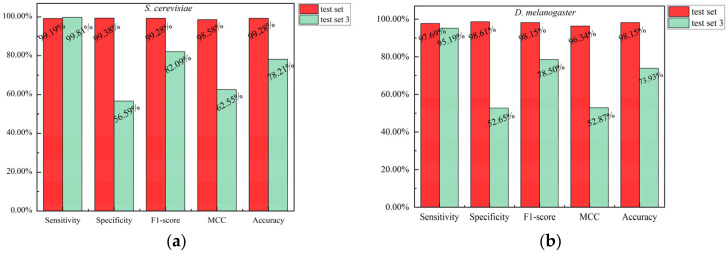

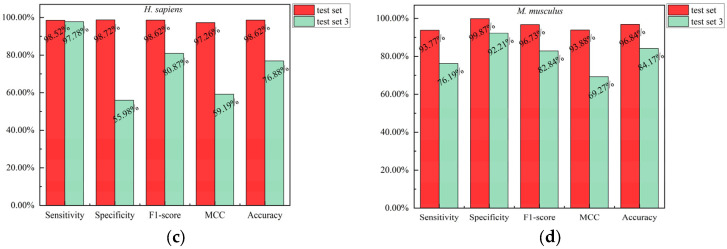
Comparison of predictions by the CNN model on test set and test set 3 across four species. (**a**) *S. cerevisiae*; (**b**) *D. melanogaster*; (**c**) *H. sapiens*; (**d**) *M. musculus*.

To evaluate the importance of constructing negative samples using the subcellular localization strategy, we used a random strategy to reconstruct negative samples and extracted them at a positive-to-negative ratio of 1:1 to generate balanced training, validation, and test sets. The predictions obtained from the random strategy on the test set were compared with those based on the subcellular localization strategy (see Figure 12). In Figure 12, we use test set 3 to represent the test set constructed using the random strategy, and test set to represent the test set constructed using the subcellular localization strategy. The optimal hyperparameter configurations for the five independent CNN models under the random strategy for each dataset (*S. cerevisiae*, *D. melanogaster*, *H. sapiens*, and *M. musculus*) are provided in Tables S9–S12.

As shown in Figure 12, when negative samples were constructed using the subcellular localization strategy, the CNN model outperformed the random strategy across all four species. On the *D. melanogaster* species, the CNN model showed the greatest difference in results between the two strategies: on the test set, specificity, F1-score, MCC, and accuracy were 98.61%, 98.15%, 96.34%, and 98.15%, respectively, while on the test set 3, these metrics were 52.65%, 78.50%, 52.87%, and 73.93%, respectively. On the *M. musculus* species, the CNN model showed a relatively small difference in results between the two strategies. This indicates that the negative samples constructed using the subcellular localization strategy in this study exhibit good compatibility with the designed CNN model, enabling more effective prediction of protein–protein interactions.

**Figure 13:**
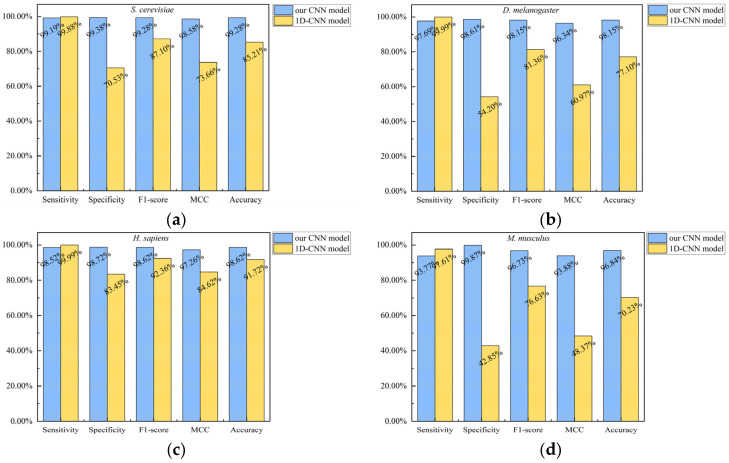
Performance comparison between our CNN model and the 1D-CNN model on the test sets of four species. (**a**) *S. cerevisiae*; (**b**) *D. melanogaster*; (**c**) *H. sapiens*; (**d**) *M. musculus*.

To evaluate the contribution of the proposed grayscale mapping strategy, we directly concatenated the extracted original features into a 1 × 1152 vector as the representation of a protein pair and employed a one-dimensional convolutional neural network (1D-CNN) model for training and prediction. Figure 13 shows a comparative analysis of the predictive performance between our constructed CNN model and the 1D-CNN model on the test sets of four species: *S*. *cerevisiae*, *D*. *melanogaster*, *H*. *sapiens*, and *M*. *musculus*.

As shown in Figure 13, compared with our CNN model, the 1D-CNN model exhibited declines in specificity, F1-score, MCC, and accuracy on the test sets of the four species (*S. cerevisiae*, *D. melanogaster*, *H. sapiens*, and *M. musculus*), while sensitivity showed a slight increase. Among these, the decrease was smallest on the *H. sapiens* and largest on the *M. musculus*. Specifically, on the *M. musculus*, the specificity dropped from 99.87% to 42.85% (a decrease of 57.02%), the F1-score dropped from 96.73% to 76.63% (a decrease of 20.1%), the MCC dropped from 93.88% to 48.37% (a decrease of 45.51%), and the accuracy dropped from 96.84% to 70.23% (a decrease of 26.61%). The above analysis indicates that the adoption of the grayscale mapping strategy helps improve the predictive performance of our model.

**Figure 14:**
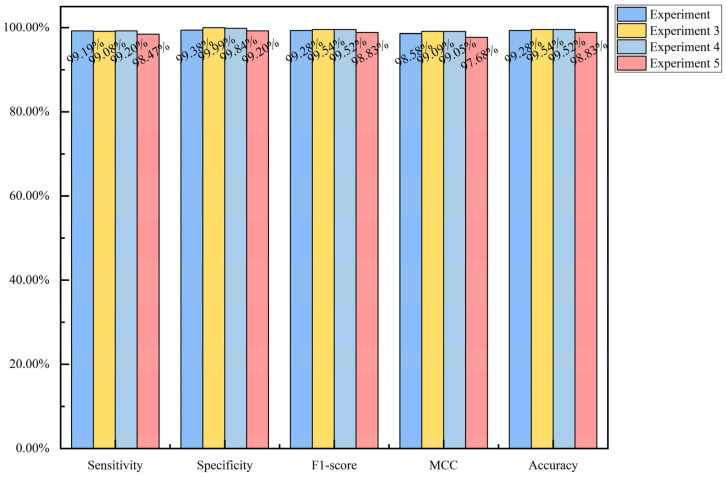
Comparison of CNN model predictions under different feature arrangements within sample feature grayscale maps in the *S. cerevisiae* species.

To investigate whether the arrangement of features in the sample feature grayscale map affects model performance, we conducted three experiments with different feature rearrangements on the *S. cerevisiae* species (see Figure 14). In Figure 14, Experiment 3, Experiment 4, and Experiment 5 represent the results of the three new rearrangement experiments, while Experiment corresponds to the experimental results of the sample feature grayscale map generated by Formula (36). The optimal hyperparameter configurations for the five independent CNN models in Experiment 3, Experiment 4, and Experiment 5 on the *S. cerevisiae* dataset are provided in Tables S13–S15. As can be seen from Figure 14, the predictions of the CNN model were largely consistent across the four experiments. This indicates that the arrangement of features in the sample feature grayscale map has almost no impact on model performance.

## 4. Discussion

Although our method demonstrated outstanding predictive performance on the test sets of all four datasets, there were still certain differences in the predictions on different test sets, although the extent of the differences was limited. Through feature importance analysis, we found that among the four types of extracted features, local same type of amino acid position variation features had a significant impact on four datasets, and the degree of influence was basically consistent, indicating that this feature type made an important contribution to both model construction and the prediction process. Secondly, the global physicochemical properties features of the protein sequence also had a significant impact on four datasets, but the degree of influence exhibited noticeable fluctuations across them, suggesting that the contribution of this feature type varied in different datasets. In contrast, the protein evolutionary conservation features and gene sequence features also contributed to the model performance, but their overall impact was relatively small.

Compared to commonly used PPI prediction methods, our method had the following advantages. (1) At the feature design level, we adopted a combination of protein sequence features and gene sequence features to characterize protein pairs. When extracting protein sequence features, we defined the same type of amino acid interval distance sequence and extracted 320 dimensional features to characterize the local same type of amino acid position variation information based on such sequences. These features were applied to protein–protein interaction prediction, and experimental results demonstrated that they significantly enhanced the predictive performance of our model. For protein evolutionary conservation features, most computational methods use the column mean ${\bar{a}}_{w}$ of the PSSM as protein evolutionary conservation features. In addition to using ${\bar{a}}_{w}$ as protein evolutionary conservation features, we also defined a mathematical descriptor $\delta_{i,w}^{\theta }$ to quantify the tendency of two amino acids at different positions to evolve into the same type of amino acid. We further extracted 60 dimensional features based on this descriptor and incorporated them with the ${\bar{a}}_{w}$ features as protein evolutionary conservation features. Experimental results demonstrated that, compared to models using only the ${\bar{a}}_{w}$ as protein evolutionary conservation features, the integrated feature $V_{3}$ effectively enhanced the overall predictive performance of the model. Subsequently, we further extracted the gene sequence features and combined them with the protein sequence features to represent protein pairs. The feature importance analysis indicated that the gene sequence features we extracted could improve the model performance. (2) In terms of feature dimensions, we used a 546 dimensional feature vector to represent a protein. Compared to the 616 dimensional features used in the AE-LGBM method and the 1280 dimensional features used in the ESMDNN-PPI method, our method achieved a lower-dimensional and more efficient protein representation. (3) In terms of model design, compared to the DeepSG2PPI, SpatialPPI, and DeepTrio methods, our constructed CNN model is more concise, comprising only three convolutional layers, two pooling layers, and a fully connected layer. In addition, through grayscale mapping strategy importance analysis, we found that converting the extracted raw features into sample feature grayscale maps to represent protein pair samples could effectively improve the predictive performance of the model, compared to directly using raw feature vectors as model input. This result indicated that our proposed grayscale mapping strategy had a significant impact on model performance.

However, our method still has certain limitations, which are primarily reflected in the following four aspects. First, the diversity of proteoforms caused by alternative splicing and post-translational modifications in the actual physiological environment has not been taken into account. These mechanisms can alter the physicochemical properties and three-dimensional structures of proteins, thereby influencing their interaction capabilities. For instance, the phosphorylation modification of microtubule-associated protein tau has been proven to have a significant impact on its functional regulation and aggregation propensity. Second, we have not yet conducted an in-depth exploration of the biological significance of the extracted features (e.g., the local same type of amino acid position variation features). Third, the negative sample construction strategy and evaluation system require further improvement. We constructed negative samples based on the subcellular localization strategy. Although this strategy is widely adopted, it still has limitations. For instance, proteins located in the same subcellular compartments may not interact, and interacting protein pairs across different subcellular compartments may be incorrectly classified as negative samples. Model evaluation was primarily conducted on balanced datasets. Although two slightly imbalanced test sets were used to validate robustness, the model performance under highly imbalanced conditions has not been systematically examined, nor have all available negative samples been fully utilized for evaluation. Fourth, the dataset split does not strictly adhere to the protein independence principle. When splitting the training, validation, and test sets, we randomly divided the data based on protein pairs, without ensuring that all interacting protein pair samples involving the same protein are assigned to the same subset. This splitting method allows the model to leverage known partial interaction information to predict the new interactions of the same protein, which has certain practical application significance. However, it also leads to an overestimation of the model performance due to protein identity leakage across subsets, affecting the accurate evaluation of its generalization ability.

In future research, improvements can be made in the following four aspects: (1) incorporating known post-translational modification information as features into the model; (2) integrating molecular dynamics simulations to introduce the native conformation and dynamic process information of proteins within cells into the model, making the study of PPIs more realistic and reliable; (3) further exploring the biological significance of the features we have extracted; (4) using multiple distinct negative sample construction strategies to make the constructed negative samples more comprehensive.

## 5. Conclusions

In this paper, we studied PPIs based on protein sequences and gene sequences, combined with CNNs. We extracted four types of features, namely the global physicochemical properties features of the protein sequence, local same type of amino acid position variation features, protein evolutionary conservation features, and the gene sequence features. Among them, the local same type of amino acid position variation features made a particularly significant contribution to the performance of the CNN model. Ablating this feature resulted in an approximately 50% performance drop in the CNN model. Our method achieved AUC values exceeding 0.95 on the test sets of four species datasets. Moreover, its successful application to the prediction of non-interacting protein pairs yielded promising results, further verifying the robustness and practical value of our method.

## Figures and Tables

**Table 1:** The results on the test sets of four species.

Species	Sensitivity	Specificity	F1-Score	MCC	Accuracy
*S. cerevisiae*	99.19% ± 0.62%	99.38% ± 0.47%	99.28% ± 0.27%	98.58% ± 0.54%	99.28% ± 0.27%
*D. melanogaster*	97.69% ± 1.49%	98.61% ± 1.70%	98.15% ± 0.95%	96.34% ± 1.90%	98.15% ± 0.95%
*H. sapiens*	98.52% ± 0.81%	98.72% ± 0.79%	98.62% ± 0.30%	97.26% ± 0.60%	98.62% ± 0.30%
*M. musculus*	93.77% ± 2.60%	99.87% ± 0.26%	96.73% ± 1.47%	93.88% ± 2.66%	96.84% ± 1.39%
Average value	97.29%	99.15%	98.20%	96.52%	98.22%

The value before the ± symbol represents the mean of the five prediction results, and the value after the ± symbol represents the standard deviation of the five prediction results.

**Table 2:** Precision of the CNN model for the top u predictions on test set 1 and test set 2.

Dataset	u = 100		u = 200		u = 300	
	Test Set 1	Test Set 2	Test Set 1	Test Set 2	Test Set 1	Test Set 2
*S. cerevisiae*	100%	100%	100%	100%	100%	100%
*D. melanogaster*	99%	100%	99.5%	100%	99%	100%
*H. sapiens*	100%	100%	100%	100%	100%	100%
*M. musculus*	100%	100%	92.02%	88.48%	92.02%	88.48%

**Table 3:** Comparison of the predictions of nine methods on the *S. cerevisiae* dataset.

Method	Sensitivity	Specificity	F1-Score	MCC	Accuracy
AE-LGBM	92.10%	98.70%	N/A *	91.00%	95.40%
OLPP-ROF	89.83%	N/A *	N/A *	82.10%	90.07%
LPBERT	N/A *	98.67%	N/A *	95.89%	97.94%
SVM-NVDT	99.03%	N/A *	99.19%	96.58%	99.20%
TAGPPI	98.26%	98.10%	97.80%	97.80%	97.81%
PPI-BAN	98.41%	98.15%	98.28%	96.56%	98.28%
SVM-FFANE	93.17%	N/A *	94.13%	88.41%	94.19%
CF-PPI	96.36%	96.12%	96.25%	92.82%	96.25%
Our method	99.19%	99.38%	99.28%	98.58%	99.28%

N/A * indicates that the corresponding reference does not provide results for this indicator.

**Table 4:** Comparison of the predictions of nine methods on the *H. sapiens* dataset.

Method	Sensitivity	Specificity	F1-Score	MCC	Accuracy
OLPP-ROF	95.20%	N/A *	N/A *	92.47%	96.09%
LPBERT	N/A *	98.58%	N/A *	97.00%	98.50%
SVM-NVDT	99.90%	N/A *	95.61%	91.19%	95.40%
SVM-FFANE	96.95%	N/A *	98.00%	95.39%	97.69%
ESMDNN-PPI	84.49%	98.81%	N/A *	84.71%	97.51%
DeepTrio	97.23%	99.01%	98.11%	96.26%	98.12%
RF-LPQ	97.32%	N/A *	N/A *	96.00%	97.96%
DeepInteract	90.47%	98.47%	N/A *	89.24%	94.47%
Our method	98.52%	98.72%	98.62%	97.26%	98.62%

N/A * indicates that the corresponding reference does not provide results for this indicator.

**Table 5:** The predictions of our method at different classification thresholds on the PPI network dataset.

Threshold	Sensitivity	Precision	F1-Score	Accuracy
0.5	100%	100%	100%	100%
0.8	100%	100%	100%	100%
0.9	100%	100%	100%	100%
0.95	100%	100%	100%	100%
0.98	100%	100%	100%	100%

**Table 6:** The predictions of our method at different classification thresholds on the PPNI network dataset.

Threshold	Specificity			Accuracy		
	One-Core Network Dataset	Multiple-Core Network Dataset	Crossing Network Dataset	One-Core Network Dataset	Multiple-Core Network Dataset	Crossing Network Dataset
0.5	100%	100%	100%	100%	100%	100%
0.2	100%	100%	100%	100%	100%	100%
0.1	100%	100%	100%	100%	100%	100%
0.05	100%	100%	100%	100%	100%	100%
0.01	100%	100%	100%	100%	100%	100%

**Table 7:** Comparison of CNN model performance using different protein evolutionary conservation features on four species.

Species	Experiment 1 (${\bar{a}}_{w}$)	Experiment 2 ($Z_{w}^{\theta }$)	Experiment ($V_{3}$)
*S. cerevisiae*	98.71% ± 0.005%	97.39% ± 0.157%	99.28% ± 0.001%
*D. melanogaster*	95.15% ± 0.053%	97.86% ± 0.040%	98.15% ± 0.009%
*H. sapiens*	97.59% ± 0.009%	98.04% ± 0.006%	98.62% ± 0.001%
*M. musculus*	92.75% ± 0.371%	94.04% ± 0.172%	96.84% ± 0.019%

The value before the ± symbol represents the mean of the five prediction results, and the value after the ± symbol represents the variance of the five prediction results.

## Data Availability

The data and code of this manuscript are available on GitHub website at: https://github.com/2583291527/PPI-CNN.git (accessed on 31 December 2025).

## Supplementary Materials

The following supporting information can be downloaded at: https://www.mdpi.com/article/10.3390/biotech15010020/s1. Table S1: The number of proteins, positive samples, and negative samples across four species. Table S2: Hydrophobicity and hydrophilicity values of each amino acid. Table S3: Optimal hyperparameter configurations of the CNN model for the *S. cerevisiae* dataset. Table S4: Optimal hyperparameter configurations of the CNN model for the *D. melanogaster* dataset. Table S5: Optimal hyperparameter configurations of the CNN model for the *H. sapiens* dataset. Table S6: Optimal hyperparameter configurations of the CNN model for the *M. musculus* dataset. Table S7: The predictions of the CNN model on test set 1 for four species. Table S8: The predictions of the CNN model on test set 2 for four species. Table S9: Optimal hyperparameter configurations of the CNN model under the random strategy for the *S. cerevisiae* dataset. Table S10: Optimal hyperparameter configurations of the CNN model under the random strategy for the *D. melanogaster* dataset. Table S11: Optimal hyperparameter configurations of the CNN model under the random strategy for the *H. sapiens* dataset. Table S12: Optimal hyperparameter configurations of the CNN model under the random strategy for the *M. musculus* dataset. Table S13: Optimal hyperparameter configurations of the CNN model in Experiment 3 on the *S. cerevisiae* dataset. Table S14: Optimal hyperparameter configurations of the CNN model in Experiment 4 on the *S. cerevisiae* dataset. Table S15: Optimal hyperparameter configurations of the CNN model in Experiment 5 on the *S. cerevisiae* dataset.
