# Supplementary material for: Predicting Protein–Protein Interactions by Convolutional Neural Network Model
Source: BioTech (Basel). 2026 Feb 16;15(1):20. doi: 10.3390/biotech15010020 (PMC12938199; doi:10.3390/biotech15010020)
Supplement: Supplementary file 1 [file biotech-15-00020-s001.zip › biotech-4084827-supplementary.pdf]

BioTech

MDPI

Article

Supplementary Materials: Predicting Protein–Protein Interactions by Convolutional Neural Network Model

Shuaibo Shi, Ting Xiong, Dong Wang, Lingling Wei, Lin Li, Zhixin Li and Yanfen Lyu

The number of proteins is 3,406 in *S. cerevisiae*, 4,596 in *D. melanogaster*, 2,347 in *H. sapiens*, and 870 in *M. musculus*. There are 3,136,614 samples in the *S. cerevisiae* dataset, of which 13,134 are interacting protein pairs, accounting for about 0.4187% of the total samples. There are 1,120,613 samples in the *D. melanogaster* dataset, of which 11,234 are interacting protein pairs, accounting for about 1.0025% of the total samples. There are 108,948 samples in the *H. sapiens* dataset, of which 3,385 are interacting protein pairs, accounting for about 3.107% of the total samples. There are 16,133 samples in the *M. musculus* dataset, of which 837 are interacting protein pairs, accounting for about 5.1881% of the total samples. The number of proteins, positive samples, and negative samples across four species is shown in Table S1. Table S2 describes hydrophobicity and hydrophilicity values of each amino acid. The optimal hyperparameter configurations of the five independent CNN models on each dataset (*S. cerevisiae*, *D. melanogaster*, *H. sapiens*, and *M. musculus*) are shown in Tables S3-S6. The predictions of the CNN model on imbalanced test set (test set 1 and test set 2) for four species are shown in Tables S7-S8. The optimal hyperparameter configurations for the five independent CNN models under the random strategy for each dataset (*S. cerevisiae*, *D. melanogaster*, *H. sapiens*, and *M. musculus*) are shown in Tables S9-S12. The optimal hyperparameter configurations for the five independent CNN models in Experiment 3, Experiment 4, and Experiment 5 on the *S. cerevisiae* dataset are shown in Tables S13-S15.

Table S1. The number of proteins, positive samples, and negative samples across four species.

Species	Number of proteins	Dataset	Positive samples	Negative samples
S. cerevisiae	3,406	training set	7,880	1,721,633
		validation set	2,627	692,011
		test set	2,627	709,836
D. melanogaster	4,596	training set	6,740	674,231
		validation set	2,247	212,151
		test set	2,247	222,997
H. sapiens	2,347	training set	2,031	56,470
		validation set	677	25,877
		test set	677	23,216
M. musculus	870	training set	501	10,353
		validation set	168	3,083
		test set	168	1,860

Table S2. Hydrophobicity and hydrophilicity values of each amino acid.

Amino acids	φ^1	φ^2
A	0.62	-0.5
C	0.29	-1.0
D	−0.9	3.0
E	−0.74	3.0
F	1.19	-2.5
G	0.48	0.0
H	−0.4	-0.5
I	1.38	-1.8
K	−1.5	3.0
L	1.06	-1.8
M	0.64	-1.3
N	−0.78	0.2
P	0.12	0.0
Q	−0.85	0.2
R	−2.53	3.0
S	−0.18	0.3
T	−0.05	-0.4
V	1.08	-1.5
W	0.81	-3.4
Y	0.62	-2.3

where φ^1 represents the hydrophobicity value of amino acid, φ^2 represents the hydrophilicity value of amino acid.

Table S3. Optimal hyperparameter configurations of the CNN model for the *S. cerevisiae* dataset.

Model	Learning Rate	Convolutional Kernels	Epochs
CNN1	0.000889	16	11
CNN2	0.000099	64	5
CNN3	0.000244	16	22
CNN4	0.000238	16	9
CNN5	0.000764	64	49

Table S4. Optimal hyperparameter configurations of the CNN model for the *D. melanogaster dataset*.

Model	Learning Rate	Convolutional Kernels	Epochs
CNN1	0.0003	16	3
CNN2	0.000125	8	17
CNN3	0.000219	32	15
CNN4	0.000318	16	15
CNN5	0.000382	8	18

Table S5. Optimal hyperparameter configurations of the CNN model for the *H. sapiens* dataset.

Model	Learning Rate	Convolutional Kernels	Epochs
CNN1	0.000472	8	22
CNN2	0.000718	64	28
CNN3	0.000102	16	28
CNN4	0.000513	32	10
CNN5	0.000248	64	24

Table S6. Optimal hyperparameter configurations of the CNN model for the *M. musculus* dataset.

Model	Learning Rate	Convolutional Kernels	Epochs
CNN1	0.000985	64	21
CNN2	0.000682	8	39
CNN3	0.000149	8	37
CNN4	0.000882	64	21
CNN5	0.000715	64	16

Table S7. The predictions of the CNN model on test set 1 for four species.

Species	Sensitivity	Specificity	F1-score	MCC	Accuracy
S. cerevisiae	99.61%	97.06%	98.36%	96.71%	98.34%
D. melanogaster	98.51%	99.63%	98.34%	98.01%	99.45%
H. sapiens	95.86%	99.52%	96.72%	96.06%	98.90%
M. musculus	89.28%	98.45%	90.63%	88.80%	96.92%

Table S8. The predictions of the CNN model on test set 2 for four species.

Species	Sensitivity	Specificity	F1-score	MCC	Accuracy
S. cerevisiae	99.12%	99.53%	97.30%	97.05%	99.50%
D. melanogaster	98.53%	99.80%	98.29%	98.11%	99.68%
H. sapiens	95.27%	99.48%	95.06%	94.56%	99.10%
M. musculus	86.90%	98.86%	87.68%	86.47%	97.78%

Table S9. Optimal hyperparameter configurations of the CNN model under the random strategy for the *S. cerevisiae* dataset.

Model	Learning Rate	Convolutional Kernels	Epochs
CNN1	0.000404	32	2
CNN2	0.000822	16	3
CNN3	0.000588	4	2
CNN4	0.000905	4	3
CNN5	0.000154	16	3

Table S10. Optimal hyperparameter configurations of the CNN model under the random strategy for the *D. melanogaster* dataset.

Model	Learning Rate	Convolutional Kernels	Epochs
CNN1	0.000135	16	1
CNN2	0.000491	8	2
CNN3	0.000331	64	1
CNN4	0.000588	32	1
CNN5	0.000898	2	10

Table S11. Optimal hyperparameter configurations of the CNN model under the random strategy for the *H. sapiens* dataset.

Model	Learning Rate	Convolutional Kernels	Epochs
CNN1	0.00082	16	6
CNN2	0.000131	32	9
CNN3	0.000948	8	9
CNN4	0.000374	16	3
CNN5	0.000075	8	65

Table S12. Optimal hyperparameter configurations of the CNN model under the random strategy for the *M. musculus* dataset.

Model	Learning Rate	Convolutional Kernels	Epochs
CNN1	0.000931	16	8
CNN2	0.000308	64	10
CNN3	0.000995	32	7
CNN4	0.000573	2	27
CNN5	0.000648	32	6

Table S13. Optimal hyperparameter configurations of the CNN model in Experiment 3 on the *S. cerevisiae* dataset.

Model	Learning Rate	Convolutional Kernels	Epochs
CNN1	0.000138	64	7
CNN2	0.000541	32	7
CNN3	0.000626	16	8
CNN4	0.000758	4	10
CNN5	0.000568	64	59

Table S14. Optimal hyperparameter configurations of the CNN model in Experiment 4 on the *S. cerevisiae* dataset.

Model	Learning Rate	Convolutional Kernels	Epochs
CNN1	0.000122	4	38
CNN2	0.00049	64	8
CNN3	0.000604	8	12
CNN4	0.000195	16	8
CNN5	0.00034	2	57

Table S15. Optimal hyperparameter configurations of the CNN model in Experiment 5 on the *S. cerevisiae* dataset.

Model	Learning Rate	Convolutional Kernels	Epochs
CNN1	0.000125	32	59
CNN2	0.00025	64	26
CNN3	0.000507	32	15
CNN4	0.000642	4	5
CNN5	0.000506	64	6
